# Address to Delinquents in the December Number

**Published:** 1894-01

**Authors:** 


### ADDRESS TO DELINQUENTS IN THE DECEMBER NUMBER.

EDITOR HOMŒOPATHIC PHYSICIAN:—I have been a faithful subscriber to your journal for many years. Its vigorous and fearless editorials have delighted and refreshed me many times when I have been weary of sham and the knowledge that "policy" rather than "truthfulness," both as regards conduct or the expression of opinion, has been more than usually distasteful to me. But the December number contained a great, I may say a painful surprise. Really, I cannot but grieve over the bad taste displayed in thus rudely calling delinquent subscribers to account.

We have gone on reading our journal year after year, in peace. The question of *paying* for the privilege has never once disturbed our equanimity. Indeed we feel that we have a *right* to our journal regularly delivered without having our soul vexed by questions of finance.

"An expense to the editor"? Nonsense! "Printers bills must be paid"? Only vulgar minds can condescend to such absurd details. Please send along my journal and leave me in peace. What! Arrears *must* be paid. Confound the fellow, I believe he means it! Such persistence is very bad form really; but as I can't do without my HOMŒOPATHIC PHYSICIAN I guess I'll have to settle up.

ONE OF THE DELINQUENTS.

